# Transient receptor potential Ankyrin‐1 (TRPA1) agonists suppress myelination and induce demyelination in organotypic cortical slices

**DOI:** 10.1002/glia.24347

**Published:** 2023-02-10

**Authors:** Vincenzo Giacco, Grace Flower, Maria Artamonova, Jake Hunter, Aitana Padilla Requerey, Nicola B. Hamilton

**Affiliations:** ^1^ Wolfson Centre for Age‐Related Diseases Institute of Psychiatry, Psychology and Neuroscience, Guy's Campus, King's College London London UK

**Keywords:** demyelination, myelination, oligodendrocytes, organotypic cortical brain slices, TRPA1

## Abstract

Oligodendrocytes are highly specialized glial cells characterized by their production of multilayer myelin sheaths that wrap axons to speed up action potential propagation. It is due to their specific role in supporting axons that impairment of myelin structure and function leads to debilitating symptoms in a wide range of degenerative diseases, including Multiple Sclerosis and Leukodystrophies. It is known that myelin damage can be receptor‐mediated and recently oligodendrocytes have been shown to express Ca^2+^‐permeable Transient Receptor Potential Ankyrin‐1 (TRPA1) channels, whose activation can result in myelin damage in ischemia. Here, we show, using organotypic cortical slice cultures, that TRPA1 activation, by TRPA1 agonists JT010 and Carvacrol for varying lengths of time, induces myelin damage. Although TRPA1 activation does not appear to affect oligodendrocyte progenitor cell number or proliferation, it prevents myelin formation and after myelination causes internodal shrinking and significant myelin degradation. This does not occur when the TRPA1 antagonist, A967079, is also applied. Of note is that when TRPA1 agonists are applied for either 24 h, 3 days or 7 days, axon integrity appears to be preserved while mature myelinated oligodendrocytes remain but with significantly shortened internodes. These results provide further evidence that TRPA1 inhibition could be protective in demyelination diseases and a promising therapy to prevent demyelination and promote remyelination.

## Main Points

- TRPA1‐eGFP mice indicate that most oligodendrocytes express TRPA1.
- Over‐activation of TRPA1 causes demyelination in organotypic brain slices.
- Chronic activation of TRPA1 prevents myelination and causes oligodendrocyte loss.

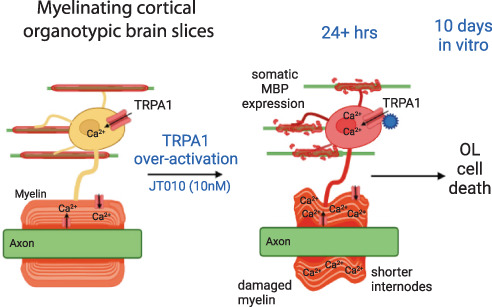

## 1 INTRODUCTION

Myelin is a specialized laminated membrane structure generated by oligodendrocytes that insulates axons and increases the action potential speed. Loss of myelin structure and function results in demyelination, that occurs in several neurological disorders including multiple sclerosis (MS), leukodystrophies, stroke, and dementia (Love, 2006; Nasrabady et al., 2018; Pendlebury et al., 2000). Currently, intense research efforts are dedicated to understanding the mechanisms causing demyelination in order to prevent myelin degeneration (Amedei et al., 2012; Desai et al., 2016; Stangel et al., 2017). Recent evidence indicates that Transient Receptor Potential Ankyrin 1 (TRPA1) channels cause myelin damage and loss of white matter function during simulated ischemia (Hamilton et al., 2016; Lajoso et al., 2021), stroke (Xia et al., 2019), and a cytotoxic model of multiple sclerosis (Bölcskei et al., 2018; Kriszta et al., 2019; Sághy et al., 2016). Previously, TRPA1 was described as a non‐selective cation channel mainly expressed in primary sensory neurons, which plays a key role in physiological and pathological pain (Chen et al., 2011; Koivisto et al., 2014, 2018; Talavera et al., 2020). TRPA1 can be activated by a wide range of exogenous molecules, including Carvacrol, allyl isothiocyanate (AITC), and JT010, and by several endogenous agents, mainly reactive oxygen, carbonyl, and nitrogen species, associated with neuroinflammatory events (Bautista et al., 2006, 2013; Hinman et al., 2006; McNamara et al., 2007). Therefore, the recent discovery of TRPA1 expression in both oligodendrocytes and astrocytes (Bosson et al., 2017; Lajoso et al., 2021; Sághy et al., 2016) highlights an important unstudied mechanism by which glial cell membrane potential and intracellular calcium concentrations can be modified, and myelin can be damaged (Hamilton et al., 2016; Lajoso et al., 2021).

Organotypic cortical brain slices provide a complex 3D in vitro model which preserves the cytoarchitecture and microenvironment of brain tissue and provides a unique tool for studying cellular development, myelination, de‐ and re‐myelination, as well as drug screening (Birgbauer et al., 2004; Hamilton et al., 2017; Rinholm et al., 2011; Yuan et al., 1998). Using this method, we have previously shown how oligodendrocyte lineage cell proliferation and myelination is regulated by endogenous GABA release (Hamilton et al., 2017).

Unfortunately, we have found that antibodies to TRPA1 were not reliable and label tissue in knockout mice. Therefore, to develop upon our functional experiments, which indicate that 100% of patch‐clamped oligodendrocytes expressed TRPA1 (Hamilton et al., 2016; Lajoso et al., 2021), and those from single cell transcriptome databases, which indicate that TRPA1 mRNA is expressed in subsets of oligodendrocytes (Marques et al., 2016), we used mice where TRPA1 expression drives EGFP production (Trpa1‐EGFP/LT325Gsat) to investigate the full extent of TRPA1 expression in oligodendrocyte lineage cells. We then confirmed using JT010, a potent site‐selective TRPA1 agonist able to activate the channel at low concentrations (EC50 = 0.65 nM; Suo et al., 2020; Takaya et al., 2015), that TRPA1 activation induces a Ca^2+^ influx into corpus callosum oligodendrocytes, and that the persistent presence of JT010 during myelination causes a failure to myelinate and loss of oligodendrocyte lineage cells, or demyelination when applied to fully myelinated tissue. As TRPA1 is known to be activated during inflammation (Bautista et al., 2006, 2013), TRPA1 mediated myelin damage and (re‐)myelination failure could be occurring in several myelin disorders and its inhibition should be considered for future therapies.

## 2 MATERIALS AND METHODS

### 2.1 Mice

C57BL/6J and transgenic TRPA1‐eGFP mice (Tg(Trpa1‐EGFP)LT325Gsat/Mmucd, MMRRC, GENSAT Project at Rockefeller University, CD1 gametes were crossed with C57BL/6J mice twice to obtain a majority (75%) C57BL6J background) of either sex were killed via schedule 1 (cervical dislocation) in accordance with the guidelines of the UK Animals (Scientific Procedures) Act 1986 and subsequent amendments.

### 2.2 Brain slice preparation for electrophysiology and single cell calcium imaging

Coronal brain slices (225 μm thick) were prepared from the brains of P12‐P17 mice in ice‐cold solution containing (mM) 124 NaCl, 26 NaHCO_3_, 1 NaH2PO_4_, 2.5 KCl, 2 MgCl2, 2 CaCl_2_, 10 glucose, bubbled with 95% O_2_/5% CO_2_, pH 7.4, as well as 1 mM Na‐kynurenate to block glutamate receptors. Brain slices were then incubated at room temperature (21–24°C) in the above solution until used in experiments. Oligodendrocytes were identified by their location and morphology. All cells were whole‐cell clamped with pipettes with a series resistance of 8–35 MΩ. Electrode junction potentials were compensated, and cells were voltage‐clamped at −74 mV.

Slices were super‐perfused with bicarbonate‐buffered solution containing (mM) 124 NaCl, 2.5 KCl, 26 NaHCO_3_, 1 NaH2PO_4_, 2 CaCl_2_, 1 MgCl_2_, 10 glucose, pH 7.4, bubbled with 95% O2 and 5% CO_2_. The flow rate was approximately 4 mL/min into a 1.5 mL bath, giving a turnover rate of under 25 s. Cells were whole‐cell clamped with electrodes containing K‐gluconate‐based solution, comprising (mM) 130 K‐gluconate, 2 NaCl, 0.01 CaCl_2_, 10 HEPES, 0.01 BAPTA, 2 NaATP, 0.5 Na_2_GTP, 2 MgCl, 10 mM phosphocreatine, and pH set to 7.15 with KOH.

Fluo‐8 (200 μM; Stratech Scientific Ltd, 21,089‐AAT) and Alexa Fluor 594 (100 μM; ThermoFischer, A10438) were used in the internal solution to measure [Ca^2+^]_i_ changes ratiometrically during experiments. Fluo‐8 and Alexa Fluor 594 fluorescence were excited sequentially using a monochromator every 3 s at 488 ± 10 nm and 585 ± 10 nm, and emission was collected using a triband filter cube (DAPI/FITC/Texas Red, 69002, Chroma Technology Corporation, Bellow Falls, VT, USA).

### 2.3 Organotypic brain cultures and brain sections

Coronal brain slices (300 μm thick) from P7 C57BL/6J mice were cultured on confetti (Millipore, FHLC01300) and basket insert (Millicell, Millipore), placed in 6‐well plates contained 1.2 mL of medium (De Simoni & Yu, 2006; Rinholm et al., 2011). The medium consists of 50% Minimal Essential Medium (MEM, GlutaMAX™ Supplement; ThermoFischer, 41090028), 23% Earl's Balanced Salt Solution (EBSS; ThermoFischer, 24010043), 25% horse serum (ThermoFischer, 26050070), penicillin and streptomycin (25 mg/mL; Sigma‐Aldrich, P0781), and 1.125% nystatin (12.5 units/mL; Sigma‐Aldrich, N1638), 36 mM d‐(+)‐Glucose solution (45% in dH_2_O; Sigma‐Aldrich, G8769), and 5 mM Tris base (Sigma‐Aldrich, 93362), at 37°C in a humidified atmosphere with 5% CO_2_. To avoid destabilizing the cultures' environment, half‐fresh medium was replaced every 3 days in both controls and treatments.

For the immunostaining of brain sections, P81‐133 Tg(Trpa1‐EGFP)LT325Gsat/Mmucd mice were culled following terminal anesthesia with sodium pentobarbital (Euthanal, 80 mg/kg, i.p.). Animals were transcardially perfused with 10% formalin and the brains post‐fixed in 10% formalin before being embedded in paraffin wax blocks. 7 μM thick coronal sections of containing the corpus collosum were cut with a microtome (Leica 710) in series, from 0.86 to 0.10 from bregma. Sections were mounted on Poly‐l‐lysine coated slides (Thermofisher).

### 2.4 Immunofluorescence, imaging, and analysis

Organotypic brain cultures were fixed with 4% paraformaldehyde (made in PBS 1x) or 10% formalin for 1 h at room temperature and washed in PBS 1x. Slices were permeabilized and blocked in PBS 1×, 5% FBS (Sigma‐Aldrich), 1% BSA (Sigma‐Aldrich), and 0.3% Triton X‐100 (Sigma‐Aldrich) at RT for 6–8 h or at 4°C overnight and then incubated over weekend at 4°C with anti‐NF160 (rabbit polyclonal, 1:200, Abcam), anti‐myelin basic protein (MBP, rat polyclonal, 1:400, Merk Millipore), anti‐Olig2 (mouse monoclonal, 1:200, Millipore, MABN50), anti‐ APC (CC1; mouse monoclonal, Merk Millipore), anti NG2 (rabbit polyclonal, 1:200 Millipore AB5320) and anti‐Ki67 (mouse monoclonal, 1:1000, BD Pharmingen 556003) primary antibodies. Subsequently, the slices were PBS‐washed and incubated with secondary antibodies diluted in blocking (without Triton X‐100) solution (without Triton X‐100) overnight at RT 4°C in the dark. The secondary antibodies were Alexa 488 goat anti‐mouse (1:500, Invitrogen), Alexa 488 goat anti‐rabbit (1:500, Invitrogen), Alexa 594 goat anti‐rabbit (1:500, Invitrogen), Alexa 555 goat anti‐rat (1:500, Invitrogen), Alexa 650 goat anti‐mouse (1:500, Invitrogen), and DAPI (Thermo Fisher Scientific). Samples were mounted on glass coverslips using mounting medium (Abcam).

To visualize TRPA1 expression, Tg(Trpa1‐EGFP)LT325Gsat/Mmucd brain sections were dewaxed using xylene and rehydrated in 100% ethanol, followed by heat‐induced antigen retrieval using citric acid (pH 6.4). Blocking was performed for 1 h with 5% bovine serum albumin (BSA) and 0.05% Triton x‐100 at room temperature. Sections were washed with TBS‐Triton x‐100 (0.05%) and incubated overnight at 4°C with primary antibody: goat anti‐ GFP (Rockland, 600‐101‐215; 1:500) and mouse anti‐Olig2, clone 211F1.1 (Sigma, MABN50; 1: 600) or mouse anti‐APC (Sigma, OP80; 1:500) or rabbit anti‐NG2 (Millipore, ab5320; 1:250) or rabbit anti‐glial fibrillary acidic protein (GFAP, DAKO, A0082; 1:1000), all dissolved in 1% BSA, 0.05% Triton x‐100: Lastly, sections were washed in TBS‐Triton x‐100 (0.05%) and incubated for 1 h with DAPI (Thermo Fisher Scientific) and donkey anti‐Goat, AlexaFluor™ 488 (Abcam, ab150129; 1:200) and donkey anti‐Mouse, AlexaFluor™ 555 (Abcam, ab150106; 1:200) or donkey anti‐rabbit AlexaFluor™ 555 (Abcam, ab150062; 1:200).

Images were acquired using a ZEISS LSM710 confocal microscope or ZEISS Axio Scan.Z1 with ×20 or oil‐immersed ×63 objective for organotypic slices and ×20 or oil‐immersed ×40 objective for brain sections. Confocal sections were acquired every 3 or 1.5 μm up to a total Z‐stack thickness of 21 μm. For each condition, we performed 2–3 independent organotypic culture experiments; from each experiment, we used 4–8 slices, and from each slice, three fields were randomly acquired. For the brain sections, we acquired the average of three images from the corpus callosum and two images from the cortex from multiple sections of 6–9 mice. Throughout the article, one slice is considered to be one “n.”

Offline analysis of the image Z‐stack was performed using the opensource image‐processing package ImageJ FIJI or QuPath measuring fluorescence intensity and counting positive or co‐localized cells manually. Myelin (myelin basic protein, MBP^+^) and axon (neurofilament, NF160^+^) process lengths were measured by making all of the images binary and then skeletonizing them with the skeleton plug‐in. All final values were normalized to the CTRL and showed as % of Control.

### 2.5 Drugs application

Stock solutions of the following drugs were made up in DMSO: Carvacrol (Sigma‐Aldrich), A967079 (Boc Sciences), and JT010 (Tocris Bioscience). When used, DMSO were also added to control at the same concentrations (normally at a dilution of or exceeding 1:1000), and did not evoke [Ca^2+^]_i_ changes or affect organotypic cultures. Stocks were kept at −20°C apart from Carvacrol which was made up fresh on each day of use. To minimize evaporation of Carvacrol, after bubbling with 95% O_2_/5% CO_2_, the lids were kept on until the solutions were used. For calcium imaging we used 2 mM of Carvacrol and 1 μM of JT010, while for organotypic cultures, 50 μM of Carvacrol, 20 μM A967079, and 10 nM of JT010 were used.

### 2.6 Statistics

For patch‐clamping experiments, one cell is an “n” and was patched in an individual cortical slice. For the brain slice experiments, all parameters measured, for example, MBP process length, NF160 intensity, Olig2+ cells, APC+ cells, and so on were averaged within a slice, and the “n” are individual cortical slices. The data include the mean ± S.E.M. *p* Values are from ANOVA tests (for normally distributed data) and Mann–Whitney *U* or Kruskal–Wallis tests (for nonparametric data). Normally distributed data were tested for equal variance (*p* < .05, unpaired *F*‐test) and multiple comparisons *p* values were corrected using the Holm–Bonferroni test. Data normality was assessed using Shapiro–Wilk tests. All statistical analysis was conducted using GraphPad Prism or OriginPro software. P values quoted in the text are from ANOVA tests unless otherwise stated.

## 3 RESULTS

### 3.1 TRPA1 is expressed in oligodendrocytes

**FIGURE 1:**
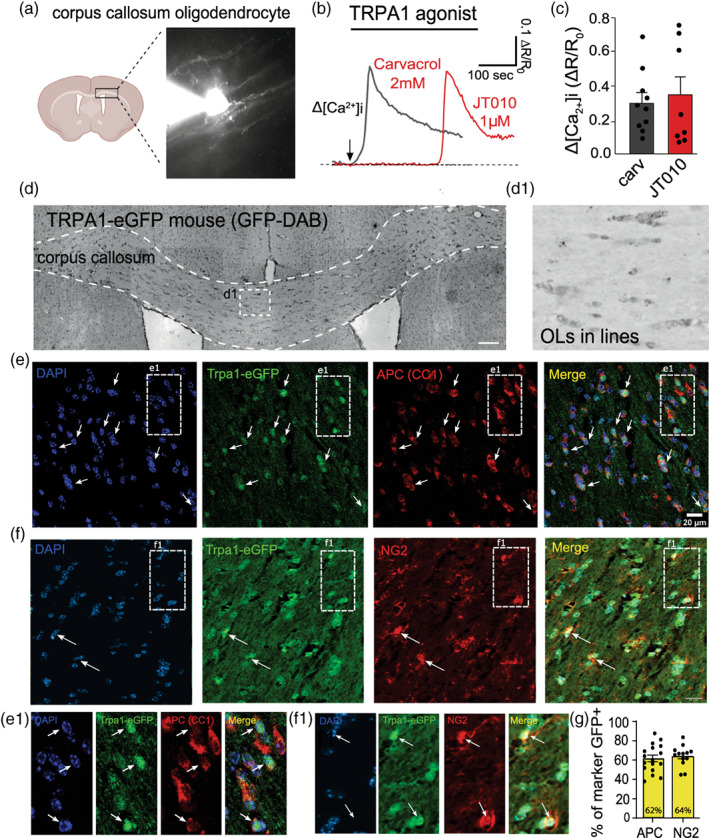
TRPA1 is expressed in oligodendrocytes. TRPA1 is expressed in corpus callosum oligodendrocytes and its activation raises [Ca^2+^]_*i*_. (a) Cartoon schematic showing the corpus callosum area with an example of a patch‐clamped oligodendrocyte filled with Fluo8 calcium dye. (b) Representative Δ[Ca^2+^]_*i*_ trace responses to TRPA1 agonists Carvacrol (gray) and JT010 (red) with (c) mean ± SEM shown in the bar plots (Δ[Ca^2+^]_*i*_ = Δ*R*/*R*_0_). “n” are cells patched in individual slices. (d) Expression of TRPA1‐eGFP was determined with DAB immunostaining of GFP. Rows of dark stained oligodendrocytes in the corpus callosum can be observed (d1). (e, f) This is confirmed with immunofluorescence labeling of DAPI (blue), eGFP‐TRPA1(green), APC (CC1) or NG2 (red), and merge (yellow). The white squares indicate the zoomed images in e1 and f which highlight colocalization of eGFP‐TRPA1(green) with either APC (CC1) or NG2(red). Arrows indicate TRPA1‐eGFP positive oligodendrocyte soma. (g) In the corpus callosum 62% of APC/CC1^+^ oligodendrocytes and 64% of NG2^+^ oligodendrocyte progenitor cells express TRPA1‐eGFP. Bar graphs are expressed as mean ± standard error of the mean. The “n” is one averaged data point from individual slices.

Using single cell calcium imaging (Hamilton et al., 2016; Lajoso et al., 2021; Figure 1a) we confirm that TRPA1 activation with either the specific TRPA1 agonist JT010 (1 μM), or the nonspecific TRPA1 agonist, carvacrol (2 mM), causes a consistent increase in intracellular calcium concentration in corpus callosum oligodendrocytes (Δ[Ca^2+^]_*i*_ = Δ*R*/*R*_0_; Figure 1b). Differences in onset times are believed to be due to variances in binding sites on TRPA1 for these two drugs (Lajoso et al., 2021), but the mean responses are the same (0.30 ± 0.04 Δ*R*/*R*_0_ Carvacrol; 0.35 ± 0.11 Δ*R*/*R*_0_ JT010; Figure 1c). To support these functional experiments and our previously published in situ hybridisation experiments showing TRPA1 mRNA in APC/CC1 positive cerebellar oligodendrocytes (Hamilton et al., 2016), we purchased TRPA1‐eGFP mice (Gensat, Tg(Trpa1‐EGFP)LT325Gsat/Mmucd) that express eGFP when TRPA1 is expressed. Initial labeling for GFP with DAB staining showed that TRPA1‐eGFP was located throughout the brain and especially in cells appearing to be oligodendrocytes in rows within the corpus callosum (Figure 1d,d1). Co‐immunofluorescence labeling for the oligodendrocyte lineage cell markers APC (CC1; Figure 1e), NG2 (Figure 1f) and Olig2 (Figure S1a) with GFP confirms that over 60% of oligodendrocyte precursors and mature oligodendrocytes express TRPA1‐eGFP in the corpus callosum (Figure 1e–g; Supporting Information Data S1). This number is reduced in the cortex where 43% of APC positive cells and 37% of olig2 positive cells express TRPA1‐eGFP (Supporting Information Data S2). Overall, approximately one third of all the cells in the white matter and gray matter express TRPA1‐eGFP (34% and 35%; Supporting Information Data S1d and S2) and 35% of GFAP positive white matter astrocytes express TRPA1 (Supporting Information Data S1b,c). TRPA1 positive cells in the white matter show similar patterns of eGFP co‐labeling, indicating that mature, immature and progenitor cells of the oligodendrocyte lineage equally express TRPA1‐eGFP. TRPA1 expression is not confined to glia in the cortex, where neuronal TRPA1‐eGFP expression can be seen (Figure S1).

### 3.2 Prolonged TRPA1 activation by JT010 affects myelin and Olig2^+^ cell number in organotypic cortical brain slices

**FIGURE 2:**
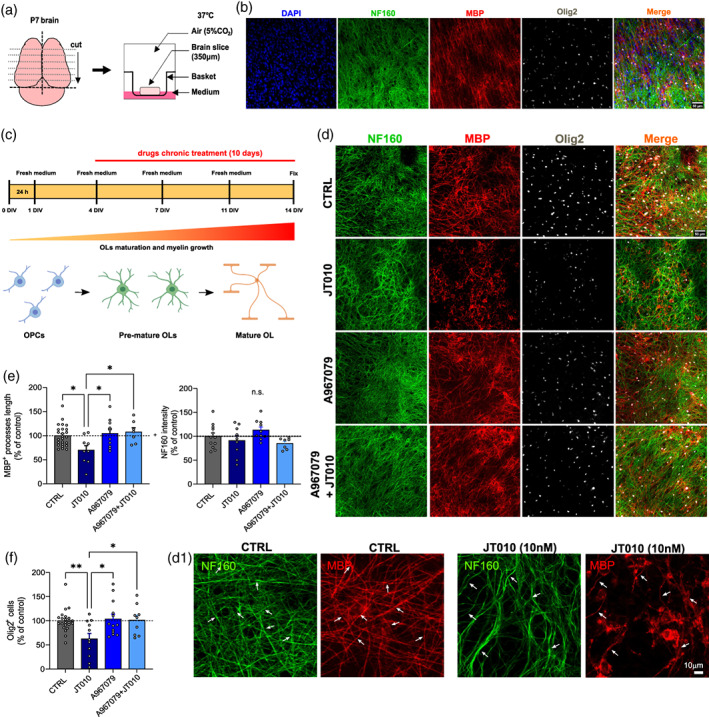
TRPA1 agonists cause myelin damage and internode shortening in organotypic cortical brain slices. (a) A cartoon schematic of the organotypic slice method in which mouse brain is sectioned (350 μm) and each individual slice is placed in basket inserts within a six‐well plate filled with culture medium. (b) After 2 weeks there is myelination, which can be determined with immunolabeling for DAPI (blue), NF160 (green), MBP (red), and Olig2 (white). (c) Over 2 weeks, with a medium change every 3 days, the oligodendrocyte precursor cells mature. (d) Representative images at DIV14 after incubation with the TRPA1 agonist, JT010 (10 nM), for 10 days shows TRPA1 mediated myelin damage that does not occur when TRPA1 is blocked with A967079 (20 μM). (d1) High power images showing the JT010 induced increase in MBP expression in oligodendrocyte soma and the change in morphology of MBP positive myelin internodes. (e) Quantification of MBP^+^ (left) internode length (% of control) in CTRL (*n* = 22), JT010 (*n* = 9), A967079 (*n* = 10), and A967079 + JT010 (*n* = 7). Quantification of NF160 fluorescence, which is unchanged. (f) Quantification of Olig2+ cells (% of control) in CTRL (*n* = 23), JT010 (*n* = 11), A967079 (*n* = 13), and A967079 + JT010 (*n* = 9). Data are mean ± SEM, with one‐way ANOVA and Bonferroni's multiple comparisons tests, *p < .05. **p < .01.

Organotypic slices are a tissue explant model with the distinctive feature of continuing to develop in vitro while maintaining the complex in vivo tissue cytoarchitecture. Thus, they are a useful method to study developmental, treatment, and disease models. Our previous work showed that organotypic cortical brain slices initially have a high number of oligodendrocyte progenitor cells (OPCs) and lower number of mature oligodendrocytes, which increase in number substantially over 2 weeks in vitro (Rinholm et al., 2011; Hamilton et al., 2017; Figure 2a,b). To determine the effect of TRPA1 activation on myelination, we incubated either the TRPA1 agonist JT010 (10 nM), the TRPA1 antagonist A967079 (20 μM), or a mix of both A967079 + JT010 from DIV 4 to DIV 14 (Figure 2c). Myelin sheaths, axons, OPCs and oligodendrocytes were visualized by MBP, NF‐160, and Olig2 co‐immunolabeling (Figure 2d,d1). Although in previous studies (Hamilton et al., 2017; Rinholm et al., 2011), we were able to use fluorescence intensity of MBP staining to measure the amount of myelin, in these cultures, it was evident that TRPA1 agonists had structurally compromised myelin (Figure 2d1) but the damaged myelin had increased fluorescence and had not been removed entirely, and therefore fluorescence intensity measurements showed no differences in most conditions (Figure S3). Therefore, to measure the obvious loss of myelin internodes, we measured MBP positive process (internode) length by skeletonisation. MBP process length was significantly decreased in JT010 (69.90% ± 9.50%; Figure 2e) compared to CTRL (*p* = .02), A967079 (*p* = 0.02), and A967079 + JT010 (*p* = 0.02; 100.00% ± 4.62%, 104.50% ± 9.49%, 107.80% ± 8.72%, respectively). Axon NF160 immunofluorescence intensity was unchanged in all conditions (Figure 2e, right). Oligodendrocyte lineage cell number, measured with Olig2 immunolabeling, significantly decreased in JT010 (62.49% ± 11.42%, *p* < .01), but not in A967079 or A967079 + JT010 (103.70% ± 9.96%, 101.10% ± 10.34%, respectively; compared to CTRL, 100.00% ± 4.78%; Figure 2f). These results suggest that TRPA1 activation causes demyelination but also may prevent myelination or remyelination.

### 3.3 OPC number and proliferation is not visibly affected by TRPA1 activation

**FIGURE 3:**
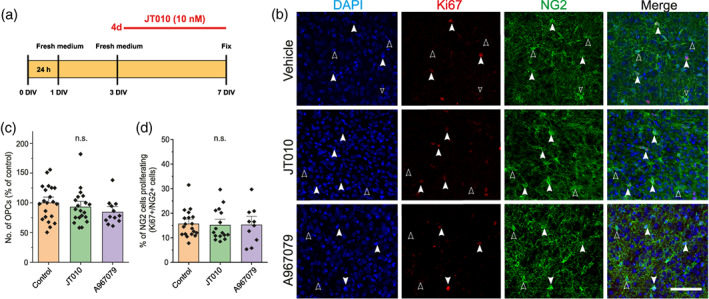
Four‐day treatment of immature cortical brain slices with JT010 does not affect OPC survival or proliferation. (a) Scheme to show the time‐course of application of the TRPA1 agonist, JT010 (10 nM), or the TRPA1 antagonist, A967079 (20 μM) to myelinating organotypic brain slices. (b) Representative images show that the number of OPCs labeled with NG2 (green), and the number of proliferating cells labeled with Ki67 (red), is unchanged in the presence of TRPA1 modulators. (c) Mean (±SEM) data showing the average number of NG2 positive cells in each slice (CTRL *n* = 21; JT010 *n* = 20; A967079 *n* = 12). (d) Mean (±SEM) data showing the percentage of NG2 cells that are Ki67 positive.

In order to determine whether TRPA1 agonist mediated loss of oligodendrocyte lineage cells is due to an effect of JT010 on OPC survival or proliferation, we incubated the myelinating cortical slices in JT010 for 4 days (DIV3 to DIV7) and then immunolabeled for OPCs with NG2‐ and dividing cells with Ki67‐ antibodies (Figure 3). We found that neither the activation of TRPA1 with JT010 (10 nM) nor the inhibition of TRPA1 with A967079 (20 μM) had any noticeable effect on OPC survival (Figure 3c) or proliferation (Figure 3d). In vehicle 7 x 10^−4^ NG2+ cells were counted per μm^2^, and 16 ± 1% of those were colabelled with Ki67. Compared to control, the number of NG2 positive OPCs was 93% ± 6% in JT010 and 84% ± 6% in A967079 (*p* > .22), and the number of OPCs proliferating was 99% ± 10% in JT010 and 99 ± 15% in A967079 (*p* = 1).

### 3.4 Acute or chronic exposure to JT010 induces myelin damage in organotypic cortical brain slices

**FIGURE 4:**
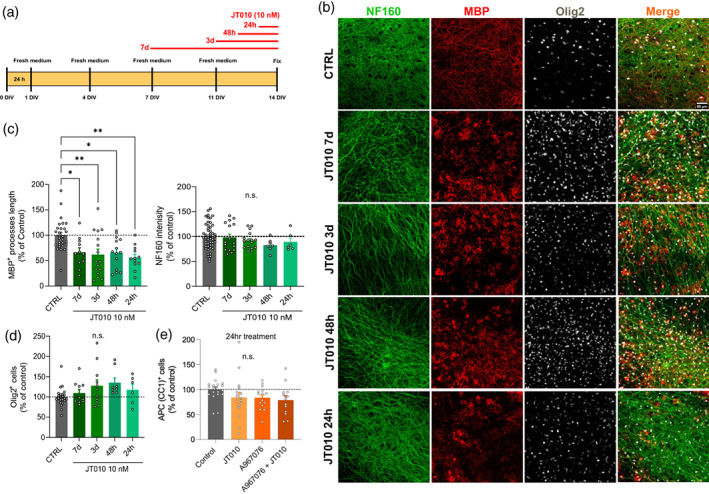
One‐day treatment with JT010 damages myelin in organotypic cortical brain slices, but oligodendrocyte loss only occurs after 7 days exposure. (a) Scheme of organotypic cortical brain slices treatment with JT010 (10 nM) for 7, 3, 2, and 1 days in vitro (DIV). (b) Representative images of 2 weeks organotypic cortical brain slice show immunolabeling for DAPI (blue), NF160 (green), MBP (red), Olig2 (white), and merge in CTRL, JT010 7 days, JT010 3 days, JT010 48 h, JT010 24 h. (c) Quantification of MBP^+^ process length (left) and NF160^+^ fluorescence intensity (right; % of control) in CTRL (*n =* 28, 25), JT010 7 days (*n =* 11, 12), JT010 3 days (*n =* 16, 10), JT010 48 h (*n =* 14, 9), JT010 24 h (*n =* 12, 9). mean ± SEM, one‐way ANOVA and Bonferroni's multiple comparisons test, *p < .05, **p < .01. (d) Quantification of Olig2+ cells (% of control) in CTRL (*n =* 23), JT010 7 days (*n =* 10), JT010 3 days (*n =* 11), JT010 48 h (*n =* 8), JT010 24 h (*n =* 6). (e) Quantification of APC/CC1 + cells (% of control) in CTRL (*n =* 19), JT010 (*n* = 17), A967079 (*n* = 15), and A967079 + JT010 (*n* = 13). Data are mean ± SEM, unpaired *t*‐test, **p < .01, ****p < .0001.

To investigate whether TRPA1 activation can affect myelin during later stages of myelination or after myelination has occurred, we incubated the organotypic slices with JT010 (10 nM) for 7 days, 3 days, 48 h, and 24 h (Figure 4a,b). In all cases, MBP positive myelin internode lengths were decreased by JT010 (7 days [66.41 ± 8.94%, *p* = .03]; 3 days [61.96% ± 10.66%, *p* = .003]; 48 h [65.88% ± 8.07%, *p* = .01]; and 24 h [56.19% ± 6.64%, *p* = .002]) compared to CTRL (100.00% ± 5.72%, Figure 4c, left), while NF160^+^ fluorescence was unchanged (Figure 4c right, *p* > .4). Contrary to the experiments where JT010 was applied for 10 days, we did not observe any significant change in Olig2^+^ cells number when it was on for shorter periods of time (Figure 4d). We confirmed that there was no change in mature oligodendrocyte number after 24 h in the presence of JT010 by immunostaining for APC/CC1, which again showed no significant change in oligodendrocyte number (CTRL [100% ± 5.05%]; JT010 24 h [83.98% ± 10.16%, *p* = .1], A969076 [83.51% ± 5.78%, *p* = .4], and A967079 + JT010 [78.76% ± 8.54%, *p* = .01; Figure 4e]).

### 3.5 Carvacrol, like JT010, induces myelin damage

**FIGURE 5:**
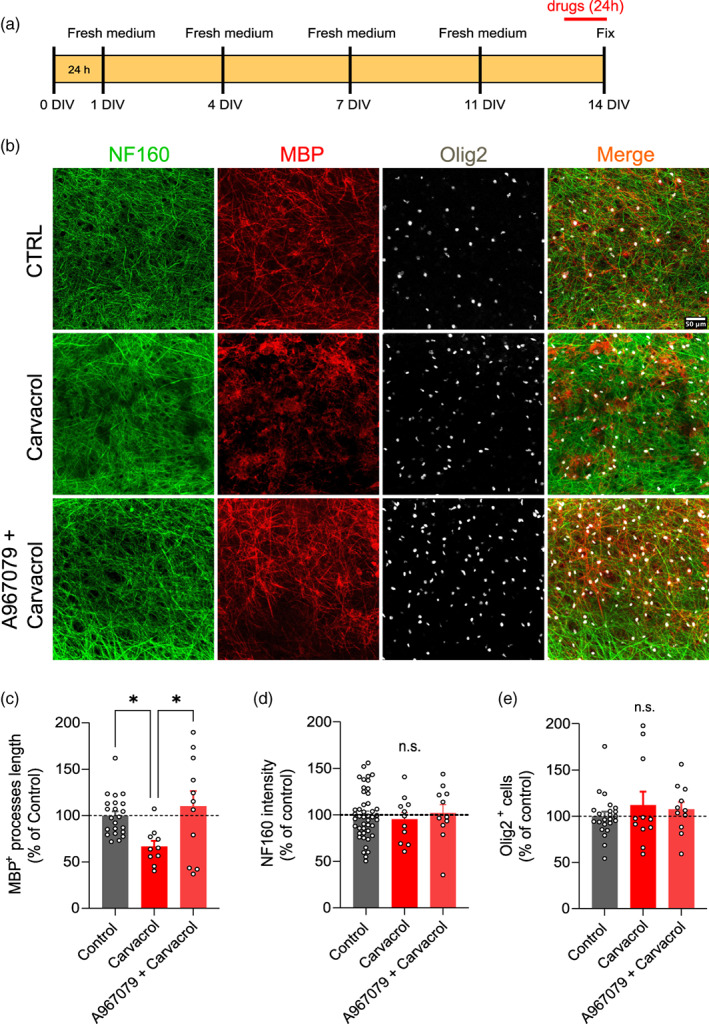
Carvacrol (24 h) induces TRPA1‐mediated demyelination in organotypic cortical brain slices. (a) Scheme of organotypic cortical brain slices treatment with Carvacrol and A967079 + Carvacrol. (b) Representative images of 2 weeks organotypic cortical brain slice show immunolabeling for DAPI (blue), NF160 (green), MBP (red), Olig2 (white), and merge in CTRL, Carvacrol, and A967079 + Carvacrol. (c) Quantification of MBP^+^ process length (% of control) in CTRL (*n* = 22), Carvacrol (*n* = 11), and A967079 + Carvacrol (*n* = 11). (d) Quantification of NF160^+^ fluorescence intensity. Data are mean ± SEM, one‐way ANOVA and Bonferroni's multiple comparisons test, *p < .05. (e) Quantification of Olig2+ cells (% of control) in CTRL (*n* = 23), Carvacrol (*n* = 11), and A967079 + Carvacrol (*n* = 11). Data are mean ± SEM, unpaired *t*‐test, **p < .01, ****p < .0001.

Several exogenous and endogenous substances have been shown to activate TRPA1. Most, like JT010, are electrophilic agonists that bind to TRPA1 on the N‐terminal by covalent modification. We, and others, have found that electrophilic TRPA1 agonists may need an intrinsic factor to be present in order to activate TRPA1 (Lajoso et al., 2021). In order to confirm that agonists that bind to TRPA1 in the traditional manner also cause demyelination, we applied carvacrol, which produces a robust TRPA1 mediated calcium concentration increase in oligodendrocytes (Figure 1). Carvacrol (50 μM) and not carvacrol + A967079 (20 μM), when applied for 24 h, caused significant demyelination, measured as decreases in MBP^+^ internode length (100% ± 4.62% CTRL; 66.78% ± 6.15% carvacrol, *p* = .03; 110.20% ± 16.37% carvacrol + A967079; Figure 5). Again, there was no significant change in NF160^+^ fluorescence (100% ± 4% CTRL; 95.30% ± 7% Carvacrol; 102% ± 9%; Figure 5d, right). Like JT010, carvacrol did not change the number of Olig2^+^ cells (100% ± 4.78% CTRL; 112.00% ± 14.16% Carvacrol; 107.5% ± 7.98%; Figure 5e), which were clearly present, but with damaged myelin.

## 4 DISCUSSION

TRPA1 is activated during periods of inflammation and oxidative stress (Bautista et al., 2013; Hamilton et al., 2016; Herrmann et al., 2019; Koivisto et al., 2014; Lennertz et al., 2012; Monteiro et al., 2020), and therefore it is important that we understand whether it plays a role in demyelinating and neurodegenerative diseases. Here, using commercially available mice that express eGFP in TRPA1 positive cells (Tg(Trpa1‐EGFP)LT325Gsat/Mmucd; MMRRC, GENSAT Project at Rockefeller University), we show that Trpa1‐eGFP colocalizes with NG2^+^,Olig2^+^ and APC(CC1)^+^ oligodendrocyte lineage cells. This evidence, along with patch‐clamping, calcium imaging, TRPA1 knockout and mRNA data (Hamilton et al., 2016; Kriszta et al., 2019; Lajoso et al., 2021; Lee et al., 2017; Marques et al., 2016; Sághy et al., 2016), show explicitly that TRPA1 is expressed in oligodendrocytes and can modulate their functions. The data presented here, and that already published, also indicate that TRPA1 is expressed by oligodendrocyte lineage cells throughout the mouse lifetime. The evidence in young mice includes (1) in situ hybridization experiments for TRPA1 mRNA (Hamilton et al., 2016; Marques et al., 2016), (2) TRPA1 agonist induced oligodendrocyte calcium flux (Hamilton et al., 2016, Lajoso et al., 2021; Figure 1), and now (3) the organotypic brain slice experiments in this article (Figures 2, 3, 4, 5). We now provide good evidence that TRPA1 continues to be expressed into adulthood by presenting data from the TRPA1‐eGFP mice (Figure 1) and this finding supports reports of TRPA1 block protecting myelin in in vivo models of stroke (Xia et al., 2019). Now it will be important to determine whether human oligodendrocytes express TRPA1 to the same degree.

Myelin is essential for speeding up the action potential and we have evidence that TRPA1 regulates neuronal excitability by controlling potassium syphoning through oligodendrocytes (observations from the Hamilton Laboratory). We believe, that TRPA1 expression is necessary for normal axonal function, as genetic loss of TRPA1, leads to reduced myelination (Lee et al., 2017) and decreased oligodendrocyte potassium conductance (observations from the Hamilton Laboratory). We find, like others, that TRPA1 is constitutively active (Karashima et al., 2010; Shigetomi et al., 2013) and regulates potassium conductance and the size of the action potential in the optic nerve (Lajoso et al., 2021). However, we have also shown that overactivation of TRPA1 during ischaemia, by raised extracellular potassium concentrations and oligodendrocyte acidification, can lead to harmful increases in intracellular calcium concentrations and separation of myelin lamellae within 1 h (Hamilton et al., 2016). Therefore, we hypothesized that over activation of TRPA1 for longer periods would lead to demyelination. Here, we have used organotypic cortical brain slices to show that direct application of TRPA1 agonists leads to demyelination, while seemingly sparing axons. Myelin damage can be seen after just 24 h, but cell loss is only observed after incubation for 10 days. Our results indicate that there is no direct effect on OPC survival and proliferation but that the presence of endogenous TRPA1 agonists would impede remyelination in demyelinating lesions occurring in Multiple Sclerosis or other neurodegenerative diseases.

Inflammation occurring in many neurodegenerative diseases, such as multiple sclerosis and Alzheimer's disease, leads to changes in the cellular environment that will favor the activation of oligodendrocyte TRPA1. Endogenous substances that increase TRPA1 expression and activity include reactive oxygen and nitrogen species (Kozai et al., 2014; Sullivan et al., 2015), hypoxia and hyperoxia (Takahashi et al., 2011), and cytokines such as interferon gamma, interleukin 1 beta and tumor necrosis factor‐alpha (Lowin et al., 2015). Environmental irritants that can cause demyelination or oligodendrocyte apoptosis (Birgbauer et al., 2004; Brambrink et al., 2012; Davies et al., 2000; Shi et al., 2011, 2015; Tian et al., 2020), have also been shown to activate TRPA1, including acrolein (Leishman et al., 2017; Park et al., 2015), toluene (Taylor‐Clark et al., 2009), lysophosphatidylcholine (Tian et al., 2020) and isoflurane (Ton et al., 2017).

As demyelination is a hallmark of every aging brain (Muñoz Maniega et al., 2015), that over years has to withstand a continuous barrage of infections, inflammation and environmental irritants, we hypothesize that overactivation of TRPA1 may play some part in the typical demyelination occurring over our lifetimes, as well as during extensive demyelination in neurogenerative diseases. In the future, the model we have generated here, can be used to dissect how oligodendrocyte TRPA1 loss, activation or inhibition affects proliferation, differentiation, demyelination, remyelination, or how TRPA1 agonists and antagonists affect microglia and astrocyte function.

Taken together, we suggest that TRPA1 block may be therapeutic in the treatment of demyelination. However, at present, we still need to determine the role of TRPA1 in the immune system and vasculature, where inconveniently, TRPA1 activation may be therapeutic (Herrmann et al., 2019; Pires & Earley, 2018; Sahoo et al., 2019; Thakore et al., 2021), and therefore the actions of TRPA1 antagonists on those cells need to be countered.

## AUTHOR CONTRIBUTIONS

Investigation, analysis, methodology, data curation, and project administration, V.G., G.F., M.A., A.P.R., J.H., and N.B.H; conceptualisation, validation, visualisation, supervision, writing, reviewing and editing, V.G., G.F. and N.B.H.; funding acquisition, N.B.H. and G.F. All authors have read and agreed to the published version of the manuscript.

## CONFLICT OF INTEREST STATEMENT

The authors declare no conflicts of interest.

## Supporting information


**Figure S1. Co‐localisation of TRPA1‐eGFP with Olig2 and GFAP in the corpus callosum.** (**a**) Immunofluorescence labeling of DAPI (blue), eGFP‐TRPA1(green), with Olig2 or (**b**) GFAP (red), and merge (yellow). White squares are zoomed images in a2 and b**2** which highlight colocalization of eGFP‐TRPA1(green) with either Olig2 or GFAP (red). Arrows indicate TRPA1‐eGFP positive oligodendrocyte or astrocyte somata. (**c**) In the corpus callosum 59% of Olig2 ^+^ oligodendrocyte lineage cells and 35% of GFAP ^+^ astrocytes express TRPA1‐eGFP. (**d**) 34% of DAPI^+^ cells express TRPA1‐eGFP in the corpus callosum (collated from all TRPA1 e‐GFP cell counts in the corpus callosum). Bar graphs are expressed as mean ± standard error of the mean. The ‘n’ is one averaged data point from individual slices.

**Figure S2. Co‐localisation of TRPA1‐eGFP and APC (CC1), NG2 or Olig2 in the cortex.** (**a**) Immunofluorescence labelling of DAPI (blue), eGFP‐TRPA1(green), APC (CC1); (**b**) NG2 or (**c**) Olig2 (red), and merge (yellow). The white squares are zoomed images in a2, b2 and c2 which highlight colocalization of eGFP‐TRPA1(green) with either APC (CC1), NG2 or Olig2 (red). Arrows indicate TRPA1‐eGFP positive oligodendrocyte soma. (**d**) In the cortex 43% of APC(CC1) ^+^ oligodendrocytes; 37% NG2 ^+^ oligodendrocyte progenitor cells and 65% of Olig2 ^+^ oligendrocyte lineage cells express TRPA1‐eGFP. (**e**) 35% of DAPI ^+^ cells express TRPA1‐eGFP in the cortex (collated from all TRPA1 e‐GFP cell counts in the cortex). Bar graphs are expressed as mean ± standard error of the mean. The ‘n’ is one averaged data point from individual slices.

**Figure S3. TRPA1 agonists and antagonist have little effect on MBP, NF160, and MBP/NF160 immunofluorescence intensity.** (**a**) After 10 days incubation with JT010 (10 nM) or A967079 (20 μM), quantification of MBP^+^ (left) and MBP/NF160 intensity (right, % of control) in CTRL (*n* = 51, 45), JT010 (*n* = 9, 9), A967079 (*n* = 10, 10), and A967079 + JT010 (*n* = 7, 7) of organotypic cortical brain slices with 10 days treatment. (**b**) Application of JT010 or A967079 for differing periods of time in vitro and quantification of MBP^+^ (left) and MBP/NF160 intensity (right, % of control) in CTRL (*n =* 51, 48), JT010 7 days (*n =* 16, 14), JT010 3 days (*n =* 16, 15), JT010 48 h (*n =* 8, 7), JT010 24 h (*n =* 6, 6). Data are mean ± SEM, one‐way ANOVA and Bonferroni's multiple comparisons test, *p < .05. (**c**) Application of the TRPA1 agonist carvacrol and quantification of MBP^+^ (left) and MBP/NF160 intensity (right, % of control) in CTRL (*n* = 51, 45), Carvacrol (*n* = 11, 10), and A967079 + Carvacrol (*n* = 11, 11).

## Data Availability

The data that support the findings of this study will be made available upon request to the corresponding author. The TRPA1‐eGFP images will be placed in an online repository for anyone to view or download.
