# Supplementary figures and images for: Transient receptor potential Ankyrin‐1 (TRPA1) agonists suppress myelination and induce demyelination in organotypic cortical slices
Source: Glia. 2023 Feb 10;71(6):1402–13. doi: 10.1002/glia.24347 (PMC10953362; doi:10.1002/glia.24347)

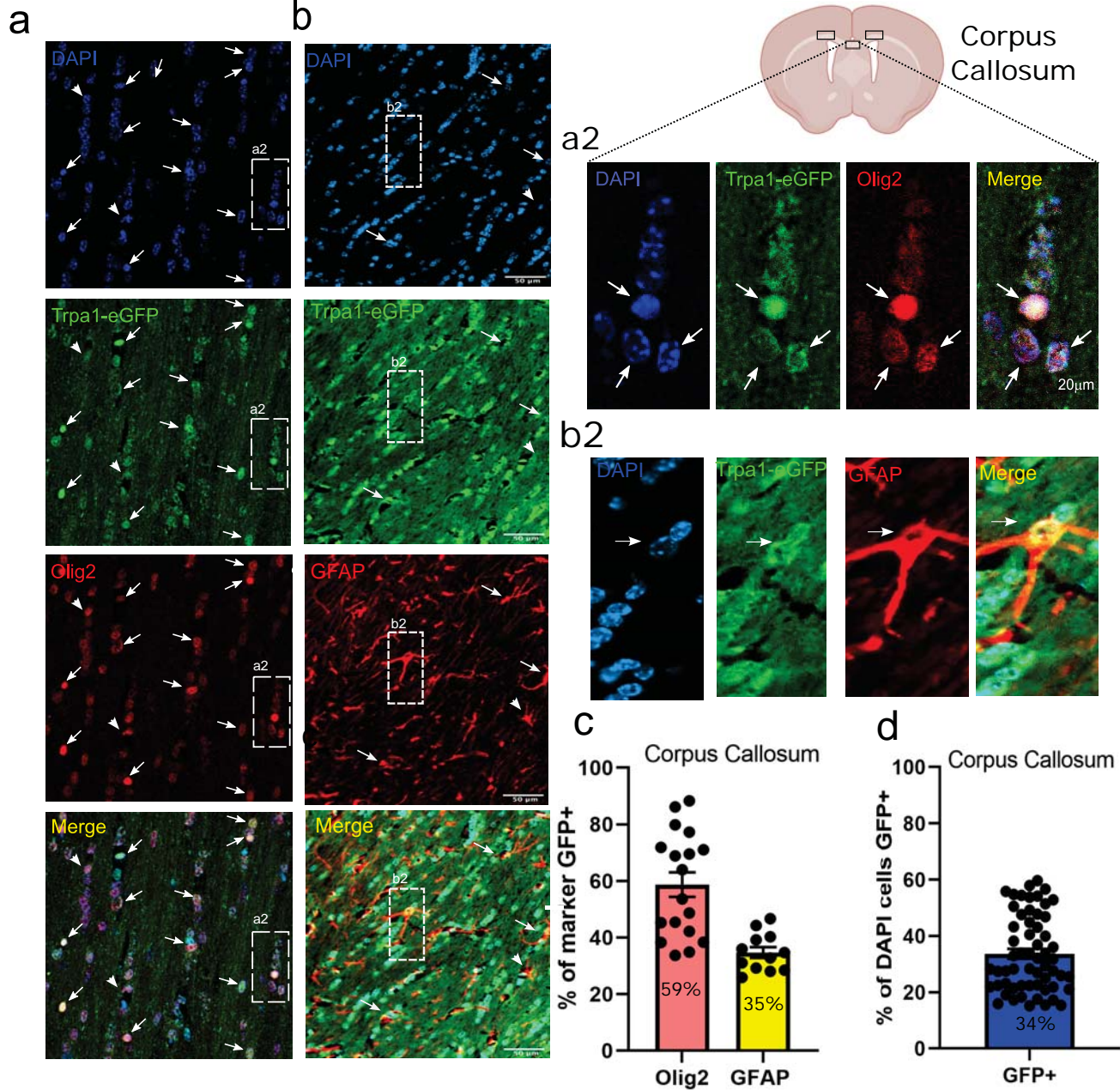

Supplement: Supplementary file 1 — Figure S1. Co‐localisation of TRPA1‐eGFP with Olig2 and GFAP in the corpus callosum. (a) Immunofluorescence labeling of DAPI (blue), eGFP‐TRPA1(green), with Olig2 or (b) GFAP (red), and merge (yellow). White squares are zoomed images in a2 and b2 which highlight colocalization of eGFP‐TRPA1(green) with either Olig2 or GFAP (red). Arrows indicate TRPA1‐eGFP positive oligodendrocyte or astrocyte somata. (c) In the corpus callosum 59% of Olig2 + oligodendrocyte lineage cells and 35% of GFAP + astrocytes express TRPA1‐eGFP. (d) 34% of DAPI+ cells express TRPA1‐eGFP in the corpus callosum (collated from all TRPA1 e‐GFP cell counts in the corpus callosum). Bar graphs are expressed as mean ± standard error of the mean. The ‘n’ is one averaged data point from individual slices. [file GLIA-71-1402-s002.pdf]

a Cortex

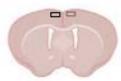
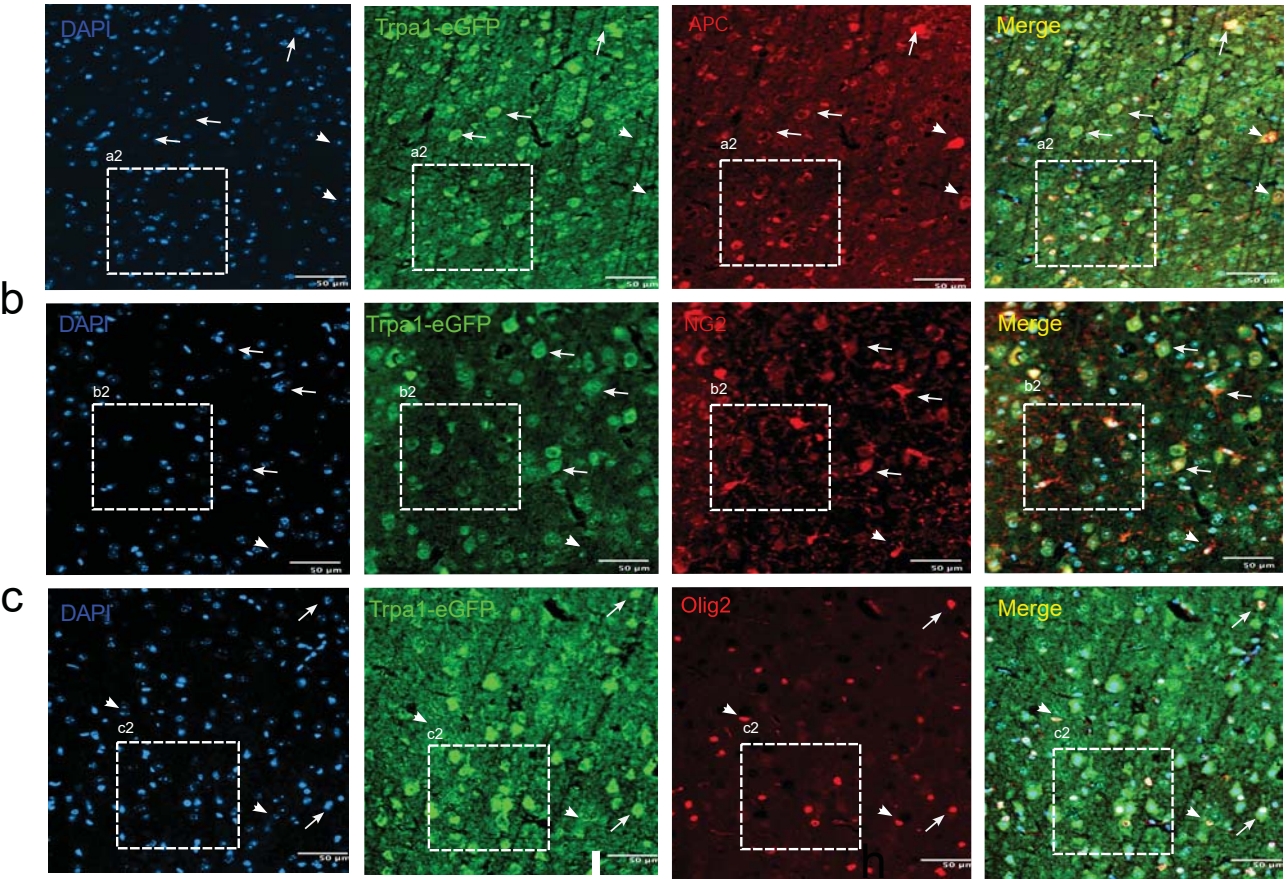

a2

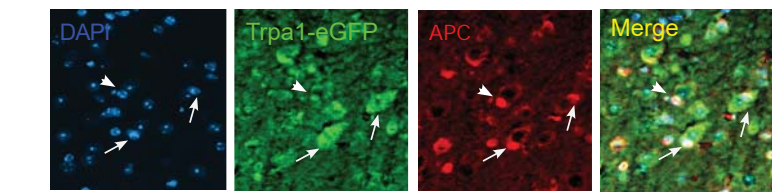

b2

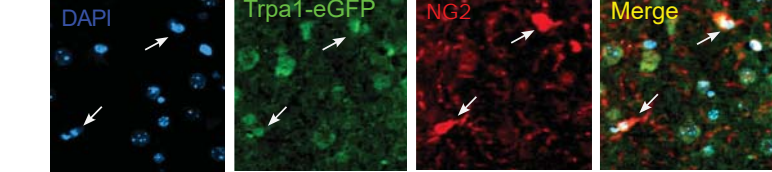

c2

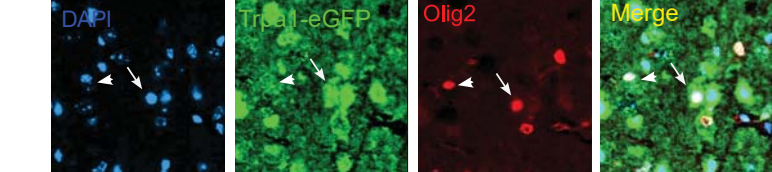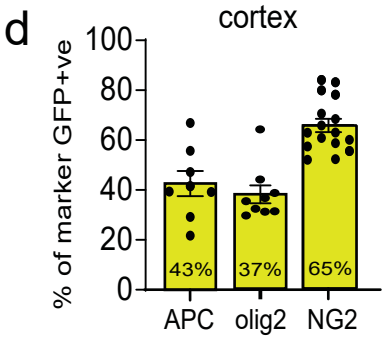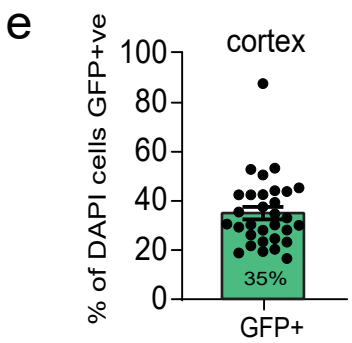

Supplement: Supplementary file 2 — Figure S2. Co‐localisation of TRPA1‐eGFP and APC (CC1), NG2 or Olig2 in the cortex. (a) Immunofluorescence labelling of DAPI (blue), eGFP‐TRPA1(green), APC (CC1); (b) NG2 or (c) Olig2 (red), and merge (yellow). The white squares are zoomed images in a2, b2 and c2 which highlight colocalization of eGFP‐TRPA1(green) with either APC (CC1), NG2 or Olig2 (red). Arrows indicate TRPA1‐eGFP positive oligodendrocyte soma. (d) In the cortex 43% of APC(CC1) + oligodendrocytes; 37% NG2 + oligodendrocyte progenitor cells and 65% of Olig2 + oligendrocyte lineage cells express TRPA1‐eGFP. (e) 35% of DAPI + cells express TRPA1‐eGFP in the cortex (collated from all TRPA1 e‐GFP cell counts in the cortex). Bar graphs are expressed as mean ± standard error of the mean. The ‘n’ is one averaged data point from individual slices. [file GLIA-71-1402-s003.pdf]

**a**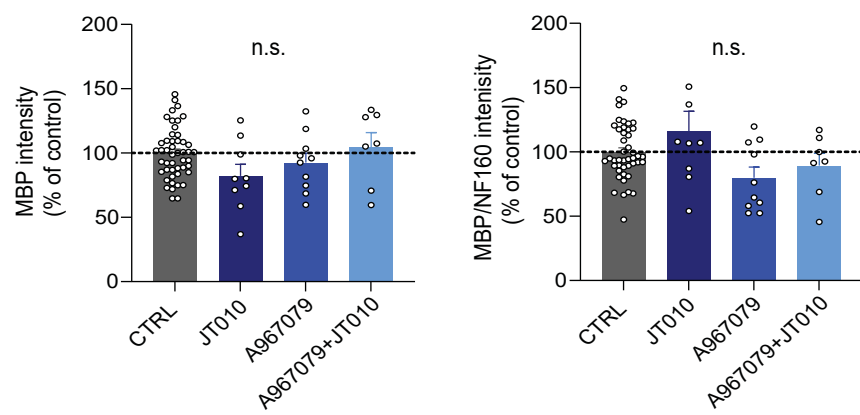**b**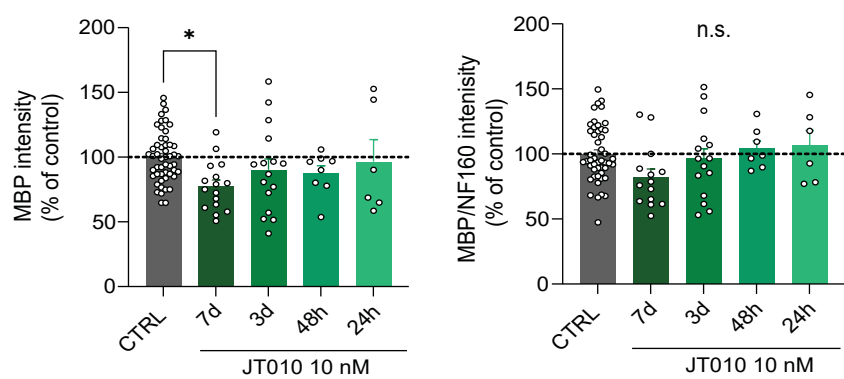**c**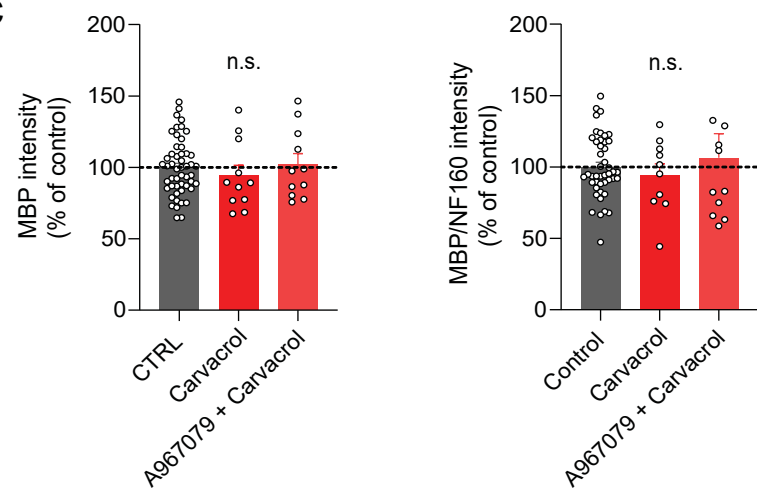

Supplement: Supplementary file 3 — Figure S3. TRPA1 agonists and antagonist have little effect on MBP, NF160, and MBP/NF160 immunofluorescence intensity. (a) After 10 days incubation with JT010 (10 nM) or A967079 (20 μM), quantification of MBP+ (left) and MBP/NF160 intensity (right, % of control) in CTRL (n = 51, 45), JT010 (n = 9, 9), A967079 (n = 10, 10), and A967079 + JT010 (n = 7, 7) of organotypic cortical brain slices with 10 days treatment. (b) Application of JT010 or A967079 for differing periods of time in vitro and quantification of MBP+ (left) and MBP/NF160 intensity (right, % of control) in CTRL (n = 51, 48), JT010 7 days (n = 16, 14), JT010 3 days (n = 16, 15), JT010 48 h (n = 8, 7), JT010 24 h (n = 6, 6). Data are mean ± SEM, one‐way ANOVA and Bonferroni's multiple comparisons test, *p < .05. (c) Application of the TRPA1 agonist carvacrol and quantification of MBP+ (left) and MBP/NF160 intensity (right, % of control) in CTRL (n = 51, 45), Carvacrol (n = 11, 10), and A967079 + Carvacrol (n = 11, 11). [file GLIA-71-1402-s001.pdf]
